# Strain-Independent Temperature Measurements with Surface-Glued Polarization-Maintaining Fiber Bragg Grating Sensor Elements

**DOI:** 10.3390/s19010144

**Published:** 2019-01-03

**Authors:** Barbara Hopf, Bennet Fischer, Thomas Bosselmann, Alexander W. Koch, Johannes Roths

**Affiliations:** 1 Munich University of Applied Sciences, Photonics Laboratory, D-80335 Munich, Germany; bennet.fischer@emt.inrs.ca (B.F.); j.roths@hm.edu (J.R.); 2 Siemens AG, Corporate Technology, CT RDA SII SSI-DE, D-91058 Erlangen, Germany; thomas.bosselmann@siemens.com; 3 Technical University of Munich, Institute for Measurement Systems and Sensor Technology, D-80290 Munich, Germany; a.w.koch@tum.de

**Keywords:** fiber optic temperature sensing, surface-attached Fiber Bragg gratings, glue-induced stress, multi-parameter measurements

## Abstract

A novel technique for strain and temperature decoupling with surface-glued fiber Bragg gratings (FBGs) is presented and applied for strain-independent temperature measurements in a temperature range between −30 °C and 110 °C with uncertainties below 4 °C over the entire measurement range. The influence of temperature-dependent glue-induced transversal forces on the fiber sensor could be eliminated with a sensor element consisting of two FBGs in identical polarization-maintaining fibers that were spliced perpendicular to each other. After aligning and gluing the sensor element with its optical axes parallel and perpendicular to the specimen, the averaged Bragg wavelength shifts of both FBGs were proven to be independent of the glue’s influence and therefore independent of any change in the material characteristics of the glue, such as aging or creeping behavior. For the first time, this methodology enables temperature measurements with surface-attached bare FBGs independently of arbitrary longitudinal and glue-induced strains. This is of great value for all applications that rely on a fully glued sensor design, e.g., in applications with high electromagnetic fields, on rotating parts, or in vacuum for space applications.

## 1. Introduction

Fiber optical temperature sensing with fiber Bragg gratings (FBGs) have gained growing importance in many industrial applications, such as temperature monitoring and temperature control of critical components [1]. Due to their advantages, such as insensitivity to high electromagnetic fields, gamma radiation, and most chemicals, a small size, and multiplexing capability, they are highly valued to replace common electrical sensors, such as thermocouples, or to enable temperature measurements in applications with harsh environmental conditions as they occur, e.g., in energy production or distribution facilities [2,3,4,5,6,7] or in space applications [8,9]. For measuring temperatures, FBGs are commonly used within a loose-tube packaging, where the FBGs are shielded from mechanical stress by a protecting capillary. Some applications, however, rely on surface-attached FBG sensing elements, which have to be glued over their entire length, especially if an air gap between the fiber and the surrounding capillary would cause severe problems. This includes all applications within high electromagnetic fields, where any gap would lead to spark discharges (e.g., hot spot detection and temperature control on the surface of the stator windings of high power generators [2]), for measuring component temperatures in vacuum, where the heat transfer from the specimen to the fiber is not sufficient (e.g., satellites in space applications [9]), or on quickly rotating components with high centrifugal forces or vibrations (e.g., monitoring of temperature distributions on turbine plates of gas turbines in power plants).

The challenges in temperature sensing with surface-glued or embedded FBG elements result from the fact that FBGs are not only sensitive to temperature but also to axial and transversal strains. If the sensor element is attached to the surface of a specimen, the FBG will therefore respond to any strain on the specimen that is caused by temperature changes or by additional external mechanical loads. Without knowledge of the exact strength of these strains, the corresponding temperature will be estimated wrongly. Therefore, FBG-based multi-parameter measurement systems, which are capable of determining temperature and strain simultaneously, are essential in these applications. Several embodiments have already been presented by different researchers [10,11], relying on the evaluation of the signals of two FBGs with different sensitivities to strain and temperature, which were located near to each other. Commonly, the fiber has been fixed at two points near the FBG-sensor elements to ensure strain transfer from the specimen to the fiber, while the sensor element itself is spared from glue. The relationship between the sensor signals (usually the Bragg wavelengths of both FBGs) and the two measurands (temperature and strain) was given by a linear system of equations expressed by a sensitivity matrix [10].

Gluing the FBG over its entire sensor length adds several challenges to the task, as the glue induces significant temperature-dependent transversal forces to the fiber, mostly because of a mismatch in the coefficients of thermal expansions (CTE) of the fiber, glue, and specimen [12,13]. These glue-induced forces alter the FBG’s sensor response, especially during temperature changes, and have to be considered in temperature measurements with surface-attached FBG elements [13,14]. This is aggravated by the fact that the used glue has to assure full strain transfer in a wide thermal range, e.g., between −40 °C and 150 °C, which requires the use of a glue with a high glass transition temperature. Thermally cured epoxy resins have been shown to fulfill this requirement, but tend to induce high values of glue-induced birefringence, especially at low temperatures [13]. Additionally, temperature-dependent creeping behavior and stress-relaxation have been observed in thermally cured epoxy resin [15,16]. Due to the relaxation characteristics of the adhesive, the amount of glue-induced stress and its influence on the FBG sensor signal varies not only with temperature but also with time, leading to a hysteresis behavior of surface-glued FBGs during temperature cycles [13]. Moreover, epoxy resins are prone to absorb humidity up to 6% of their mass, causing a swelling of the glue depending on their chemical composition, the exposure time, and the concentration [17,18]. This volume change would also lead to glue-induced stress in the fiber core, adding to the thermal-induced stress, again leading to time-dependent glue-induced wavelength shifts and therefore uncertainties in the measurement.

The solution to overcome this problem would be using a multi-parameter measurement system consisting of four FBG sensor signals with different sensitivities to temperature and the longitudinal and two transversal normal strains in the fiber. In this case, the corresponding sensitivity matrix would consist of 16 independent matrix elements [19]. Azimuthally aligned FBGs in polarization-maintaining fibers (PM-FBGs) have been proposed for measuring transversal line forces along the optical fiber axis [20]. To determine three-axial normal strains plus temperature, PM-FBGs have been combined with another technique for temperature and axial strain decoupling, such as the dual wavelength method [19,21]. Two superimposed PM-FBGs with different grating periods [19,21], for example, led to a set of four spectral peaks with slightly different sensitivities. Since their sensitivities are nearly similar, the corresponding matrix is not well-conditioned, and all work presented so far reduced the evaluated set of parameters to three in order to reduce uncertainties in the measurement, e.g., to three normal strains at isothermal temperatures [19]. The work of Abe [21] excluded one of the two transversal loads and measured longitudinal strain and transversal strain in direction of the fibers’ fast axis for measurement at two temperatures, 15 °C and 45 °C, which still is a very limited temperature range. The uncertainties in the temperature measurement were 10 °C. Therefore, until now, a satisfying temperature measurement technique with surface-glued fibers has not been available.

Here, we present a new approach using surface-attached PM-FBG elements for temperature measurements in the presence of arbitrary longitudinal external strains, which is additionally capable of compensating for glue-induced transversal forces independently of their strengths. The concept is based on decoupling temperature and strain by using the signals of the slow and the fast axes of two PM-FBGs. A second PM-FBG in the same type of fiber, which is spliced with its slow axis aligned to the fast axis of the first fiber, is used for transversal strain compensation. Orientating the optical axes (slow and fast axis) of the fiber parallel and perpendicular to the surface of the specimen ensures that the mechanical normal strains act only in the directions of the optical fiber axes. This investigation shows that for the slow and fast fiber axes, respectively, the averaged Bragg wavelengths of both FBGs were almost independent of the glue’s influence. The multi-parameter problem can therefore be reduced to two influencing variables—temperature and longitudinal strain—and the sensitivity matrix to four independent matrix elements. Nonlinearities in the temperature characteristics of FBGs are commonly known, and have also to be integrated in the evaluation process by introducing temperature-dependent matrix elements in the sensitivity matrix. The determination of strain and temperature is performed iteratively with the method presented in [22]. For the first time, to our knowledge, the here-presented technique enables temperature measurements with surface-glued FBGs independently of external longitudinal strains.

## 2. Theory: Surface-Attached FBGs for Temperature Sensing

**Figure 1:**
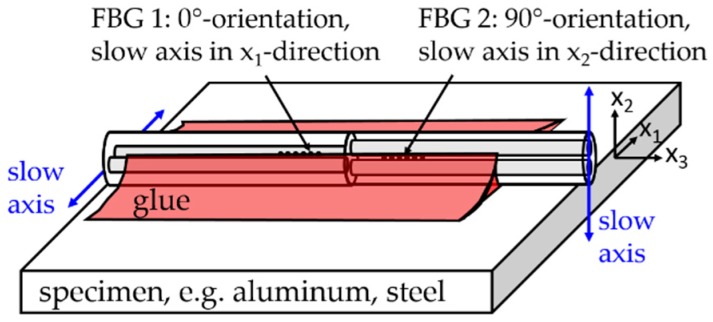
A surface-attached polarization-maintaining fiber Bragg grating (PM-FBG) tandem sensor element for strain-decoupled temperature measurements: The sensor tandem consists of a splice of a Panda fiber with its slow axis aligned perpendicular to the slow axis of the second fiber. Two FBGs were inscribed at either side of the splicing joint. The sensor tandem is azimuthally aligned with the slow and fast axis parallel and perpendicular to the surface and glued along the whole length of the sensor element to the specimen.

The PM-FBG sensor element (FBG tandem) consists of two FBGs with different Bragg wavelengths located at each side of a splice joint between standard Panda fibers (PM1550-XP, Nufern, East Granby, CT, USA). The slow axis of one fiber was aligned perpendicular to the slow axis of the other. This “FBG tandem” is glued with the optical fiber axes parallel and perpendicular to the surface, as depicted in Figure 1. The slow axis of FBG 1 is aligned parallel to the surface in the direction of $x_{1}$, referred to as the 0°-orientation ($j=0^{{}^{\circ}}$), while the slow axis of FBG 2 is aligned in the direction of $x_{2}$, referred to as the 90°-orientation ($j=90^{{}^{\circ}}$). This assures that all glue-induced normal strains in the fiber core act only in direction of the slow and fast axis. Shear strains in the fiber core and therefore mode coupling between the two polarizations may be neglected in further considerations.

In general, the spectrum of a PM-FBG is characterized by two Bragg peaks, for light polarized in the direction of the fast and the slow fiber axis, respectively. The corresponding Bragg wavelengths are given by $\lambda_{i}=2n_{\mathrm{eff},i}\Lambda$ (with i∈{s,f}, indicating the values originating from the fast and slow axis of the FBG). Here, $n_{\mathrm{eff},i}$ is the effective refractive index and Λ is the grating period, both depending on temperature and strain. This leads to changes of the Bragg wavelengths, as given by [23]

(1) $$\begin{array}{c} \Delta \lambda_{s}=\lambda_{0}((\xi +\alpha )\Delta T-\frac{n_{\mathrm{eff},s,0}}{2}(p_{11}\varepsilon_{s}+p_{12}(\varepsilon_{3}+\varepsilon_{f}))+\varepsilon_{3}),\\ \Delta \lambda_{f}=\lambda_{0}((\xi +\alpha )\Delta T-\frac{n_{\mathrm{eff},f,0}}{2}(p_{11}\varepsilon_{f}+p_{12}(\varepsilon_{3}+\varepsilon_{s}))+\varepsilon_{3}), \end{array}$$

where ξ describes the thermal optical coefficient, $p_{11}$ and $p_{12}$ the Pockel’s coefficients, and α the coefficient of thermal expansion (CTE) of the fiber; $\varepsilon_{i}$ (with i∈{f,s,3}) are the normal strains in the fiber’s core in the directions of the fast, slow, and longitudinal fiber axes; and $n_{\mathrm{eff},i,0}$ (with i∈{f,s}) are the effective refractive indices in the slow and the fast axis without strains.

The shift of Bragg wavelengths of the surface-glued sensor, $\Delta \lambda_{i,j}=\Delta \lambda_{i,j}^{\mathrm{glue}}+\Delta \lambda_{i,j}^{\mathrm{th}}+\Delta \lambda_{i,j}^{\mathrm{mech}}$ (with i∈{f,s}, indicating the values from the fast and slow axis of the FBG in the 0°- and 90°-orientation given by $j\in \{0^{{}^{\circ}},90^{{}^{\circ}}\}$), may be expressed as a sum of the glue-induced changes $\Delta \lambda_{i,j}^{\mathrm{glue}}$ due to transversal forces adding to the changes caused by temperature $\Delta \lambda_{i,j}^{\mathrm{th}}$ and external longitudinal strain $\Delta \lambda_{i,j}^{\mathrm{mech}}$. In a simplified approach, the glue-induced transversal forces can be described as external temperature-dependent line forces along the optical fiber axis, summarized in a parameter $f_{\mathrm{glue}}(\Delta T,t)$, which, due to the creeping behavior or water absorption of adhesives, also depend on time. Therefore, their strength varies constantly leading to unknown wavelength changes $\Delta \lambda_{i,j}^{\mathrm{glue}}$ that cannot be predicted in a real-world application and that, therefore, have to be compensated for in the sensor evaluation in order to avoid uncertainties in the measurement.

Assuming linear dependency, the total change of Bragg wavelength is given in the form

(2) $$\Delta \lambda_{i,j}=K_{f_{\mathrm{glue}},i,j}f_{\mathrm{glue}}(\Delta T,t)+\underset{K_{T,i,j}^{\mathrm{gl}}}{\underset{︸}{\left(K_{T,i,j}+\alpha_{\mathrm{sub}}K_{\varepsilon_{3},i,j}\right)}}\Delta T+K_{\varepsilon_{3},i,j}\varepsilon_{3},$$

where $K_{T,i,j}$ and $K_{\varepsilon_{3},i,j}$ are the temperature and strain sensitivities of the unglued PM-FBGs, respectively, and $\alpha_{\mathrm{sub}}$ is the CTE of the substrate material. Assuming full strain transfer from the substrate to the fiber, the temperature sensitivity of the glued fiber is given by $K_{T,i,j}^{\mathrm{gl}}=K_{T,i,j}+\alpha_{\mathrm{sub}}K_{\varepsilon_{3},i,j}$. Additionally, $K_{f_{\mathrm{glue}},i,j}$ expresses the influence of the glue-induced line forces to the FBG during temperature changes.

Finding sensor signals, which are independent of glue-induced transversal stresses, is a main precondition for utilizing surface-attached PM-FBGs in strain and temperature decoupling. Earlier experimental evaluation with surface-attached PM-FBGs [24] indicated that the glue-induced changes $\Delta \lambda_{i,j}^{\mathrm{glue}}$ are much stronger in the slow axis compared to the fast axis, but run counter in both FBG-orientations with $K_{f_{\mathrm{glue}},i,0^{{}^{\circ}}}\approx -K_{f_{\mathrm{glue}},i,90^{{}^{\circ}}}$ (with i∈{f,s}). Therefore, the mean value of the Bragg wavelength changes in the slow and fast axis of both sensors $\Delta \bar{\lambda_{i}}=0.5\left(\Delta \lambda_{i,0^{{}^{\circ}}}+\Delta \lambda_{i,90^{{}^{\circ}}}\right)$ with i∈{f,s} are supposed to be nearly independent of the glue’s influence, as was confirmed by the three-dimensional finite element method (3D-FEM) simulations shown in Section 2. The changes of the mean wavelength of both FBG $\Delta \bar{\lambda_{i}}$ to strain and temperature are almost independent of the glue and given by

(3) $$\Delta {\bar{\lambda }}_{i}≅{\bar{K}}_{T,i}^{\mathrm{gl}}\Delta T+{\bar{K}}_{\varepsilon_{3},i}\varepsilon_{3}.$$

The equation system for strain and temperature decoupling may therefore be expressed by means of the sensitivity matrix in the form

(4) $$\left(\begin{array}{l} \Delta {\bar{\lambda }}_{f}\\ \Delta {\bar{\lambda }}_{s} \end{array}\right)=\left(\begin{array}{cc} {\bar{K}}_{T,f}^{\mathrm{gl}} & {\bar{K}}_{\varepsilon_{3},f}\\ {\bar{K}}_{T,s}^{\mathrm{gl}} & {\bar{K}}_{\varepsilon_{3},s} \end{array}\right)\left(\begin{array}{l} \Delta T\\ \varepsilon_{3} \end{array}\right).$$

Here, ${\bar{K}}_{T,i}^{\mathrm{gl}}=0.5\left(K_{T,i,0^{{}^{\circ}}}^{\mathrm{gl}}+K_{T,i,90^{{}^{\circ}}}^{\mathrm{gl}}\right)$ and ${\bar{K}}^{\varepsilon_{3},i}=0.5\left(K_{\varepsilon_{3},i,0^{{}^{\circ}}}+K_{\varepsilon_{3},i,90^{{}^{\circ}}}\right)$ are the mean temperature and strain sensitivity constants of the two FBGs in the surface-attached FBG tandem. Temperature and strain values are evaluated by using the inverted matrix in the form

(5) $$\left(\begin{array}{l} \Delta T\\ \varepsilon_{3} \end{array}\right)=\frac{1}{D}\left(\begin{array}{cc} {\bar{K}}_{\varepsilon_{3},s} & -{\bar{K}}_{\varepsilon_{3},f}\\ -{\bar{K}}_{T,s}^{\mathrm{gl}} & {\bar{K}}_{T,f}^{\mathrm{gl}} \end{array}\right)\left(\begin{array}{l} \Delta {\bar{\lambda }}_{f}\\ \Delta {\bar{\lambda }}_{s} \end{array}\right)$$

with $D={\bar{K}}_{\varepsilon_{3},f}{\bar{K}}_{T,s}^{\mathrm{gl}}-{\bar{K}}_{T,f}^{\mathrm{gl}}{\bar{K}}_{\varepsilon_{3},s}$ indicating the determinant of the sensitivity matrix.

Assuming a linear change in the Bragg wavelength with temperature is only valid in a small temperature range. In the extended temperature range between −40 °C and 150 °C, the change in Bragg wavelength is commonly expressed with a 3rd order polynomial function [22,25]. Using this approach, the mean wavelength can be written as

(6) $$\begin{array}{cc} \Delta {\bar{\lambda }}_{i} & =a_{T,i}\Delta T+a_{T^{2},i}\Delta T^{2}+a_{T^{3},i}\Delta T^{3}\\ & =\underset{{\bar{K}}_{T,i}^{\mathrm{gl}}(\Delta T)}{\underset{︸}{\left(a_{T,i}+a_{T^{2},i}\Delta T+a_{T^{3},i}\Delta T^{2}\right)}}\Delta T. \end{array}$$

This would lead to systematic errors in temperature sensing if this nonlinearity was neglected in a large temperature range. The nonlinear sensor characteristic may be taken into account by replacing the constant matrix elements in the sensitivity matrix with temperature-dependent sensitivity constants ${\bar{K}}_{T,i}^{\mathrm{gl}}(\Delta T)$ and using the iterative calculation method introduced in [22]. Starting with the sensitivity values for a reference temperature $T_{0}$, the temperature values of the previous iteration step $\Delta T^{k-1}$ and the corresponding sensitivity values ${\bar{K}}_{T,i}^{\mathrm{gl}}{\left(\Delta T\right)}^{k-1}$ are used to calculate new temperature values $\Delta T^{k}$ according to

(7) $$\left(\begin{array}{l} \Delta T^{k}\\ \varepsilon_{3}^{k} \end{array}\right)=\frac{1}{D{\left(\Delta T\right)}^{k-1}}\left(\begin{array}{cc} {\bar{K}}_{\varepsilon_{3},s} & -{\bar{K}}_{\varepsilon_{3},f}\\ -{\bar{K}}_{T,s}^{\mathrm{gl}}{\left(\Delta T\right)}^{k-1} & {\bar{K}}_{T,f}^{\mathrm{gl}}{\left(\Delta T\right)}^{k-1} \end{array}\right)\left(\begin{array}{l} \Delta {\bar{\lambda }}_{f}\\ \Delta {\bar{\lambda }}_{s} \end{array}\right)$$

with the temperature-dependent determinant of the sensitivity matrix given by $D{\left(\Delta T\right)}^{k-1}={\bar{K}}_{\varepsilon_{3},f}\cdot {\bar{K}}_{T,s}^{\mathrm{gl}}{\left(\Delta T\right)}^{k-1}-{\bar{K}}_{\varepsilon_{3},s}\cdot {\bar{K}}_{T,f}^{\mathrm{gl}}(\Delta T)$. The iteration process is continued until the difference of the actual temperature and that of the previous iteration step drops below a certain predefined threshold value, e.g., of 0.1 °C, which is normally the case after about five iteration steps.

## 3. Simulation: Determination of Glue-Independent FBG Signals

**Figure 2:**
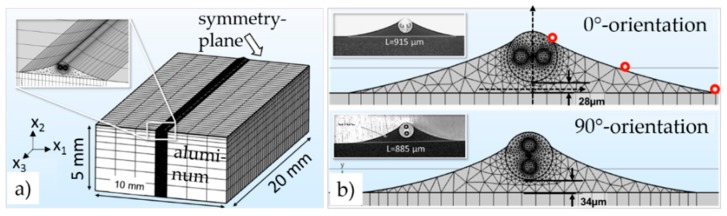
The used mesh and gluing joints for the mechanical three-dimensional finite element method (3D-FEM) simulation: (**a**) An overview of the aluminum specimen, glue, and PM fiber; (**b**) A detailed view of the geometry of the gluing joints. The insets show the metallurgic cross-sections of the glued PM-FBG after curing at 150 °C for one hour.

As discussed in the previous section, finding appropriate glue-independent sensor outputs for temperature and strain decoupling was a primary task. Therefore, the glue’s influence on the sensor signal was evaluated in detail with a three-dimensional mechanical finite element method (FEM) model capable of determining the full stress tensor at every node in the model of a glued PM fiber for different temperatures. In the model, the PM fiber with core, cladding, and stress-applying parts (SAPs), the gluing joints, and the aluminum specimen on which the fiber was glued were taken into account (see Figure 2a,b). The simulated thermally induced local stress values were used to analytically calculate the resulting changes of the refractive index caused by the elasto-optical effect. These local changes in the refractive index were added to the former values of the refractive index at the reference temperature [24].

The transfer to the effective refractive index of the fiber $\Delta n_{\mathrm{eff},i,j}^{\mathrm{sim}}(\Delta T,\varepsilon_{3})$, a quantity that is directly connected to the Bragg wavelength of the FBG, was achieved by using a two-dimensional mode solver on the fiber’s cross-section in the middle of the fiber. The resulting simulated change in the Bragg wavelength $\Delta \lambda_{i,j}^{\mathrm{sim}}$ was determined analytically by using the dependency [24]

(8) $$\Delta \lambda_{i,j}^{\mathrm{sim}}=\lambda_{0}\left(\varepsilon_{3}^{\mathrm{sim}}+\frac{\Delta n_{\mathrm{eff},i,j}^{\mathrm{sim}}(\Delta T,\varepsilon_{3})}{n_{\mathrm{eff},0}}+\xi \;\Delta T\right).$$

Here, $\varepsilon_{3}^{\mathrm{sim}}=\varepsilon_{3}^{\mathrm{mech}}+\varepsilon_{3}^{\mathrm{th}}$ describes the simulated strain in the $x_{3}$-direction at the center of the fiber’s cross section, to which both the thermal strain $\varepsilon_{3}^{\mathrm{th}}$ and the external strain $\varepsilon_{3}^{\mathrm{mech}}$ contribute. $\xi =6.36\cdot 10^{-6{}^{\circ}}C^{-1}$ is the thermo-optical coefficient of the fiber [22] and $\Delta n_{\mathrm{eff},i,j}^{\mathrm{sim}}(\Delta T,\varepsilon_{3})=n_{\mathrm{eff},i,j}^{\mathrm{sim}}(\Delta T,\varepsilon_{3})-n_{\mathrm{eff},0}^{\mathrm{sim}}$ (with i∈{s,f} and $j\in \{90^{{}^{\circ}},0^{{}^{\circ}}\}$) is the simulated change in the effective refractive index, given as the difference of the effective refractive index after temperature changes and the initial refractive index $n_{\mathrm{eff},0}^{\mathrm{sim}}$ of the stress-free model.

The FEM model describes a PM fiber glued on an aluminum specimen 5 mm in height, 10 mm in width, and 20 mm in length, with a symmetry plane at one end and a free surface at the other as pictured in Figure 2a. The geometry of the gluing joints pictured in Figure 2b was taken according to the geometries observed in metallurgic cross-sections (see Figure 2b inset) after gluing with an isothermal curing process at 150 °C. A spline plot through three characteristic points (red dots in Figure 2b) on the glue’s surface was used to define the glue’s geometry in the FEM model. The material properties of the glue (EP353, Epoxy Technology Inc., Billerica, MA, USA), which are Young’s modulus, Poisson’s ratio, and CTE, were taken from manufacturer data sheets [26,27]. The geometry and material parameters of the fiber core, SAP, and cladding were taken according to previous works [24]. All used material properties are summarized in Table 1. The simulation was performed for six steps between 110 °C and −40 °C with a step size of 30 °C.

Additionally, it was assumed that significant glue-induced forces occur only below 110 °C, which is the beginning of the glue’s glass transition range [13,28]. Therefore, all Bragg wavelength changes were calculated with respect to the reference temperature of 110 °C, at which the model was considered to be stress-free.

A second simulation was used to estimate the expected temperature-dependent wavelength shifts $\Delta \lambda_{i}^{\mathrm{theor}}$ for the fast and slow axis of an FBG, which is not exposed to any transversal glue-induced stress while the (thermal) strain is fully transferred from the aluminum specimen to the fiber during temperature changes. A similar fiber model but without glue and the aluminum specimen was applied for this purpose, in which the front cross-section of the fiber was deflected to strain values of $\varepsilon_{3}=\alpha_{\mathrm{Alu}}\Delta T$ at every simulated temperature. This displacement is equivalent to the thermal strain induced by the aluminum specimen.

**Figure 3:**
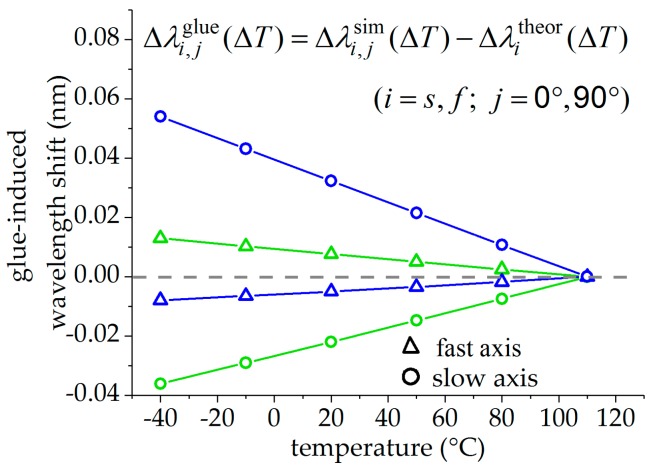
Simulated temperature-dependent deviation of the Bragg wavelength changes of the glued fiber and the theoretical transversal load-free FBG: Green are the values of the FBG in the 90°-orientation, blue of the FBG in the 0°-orientation, triangles of the fast and circles of the slow axis.

**Figure 4:**
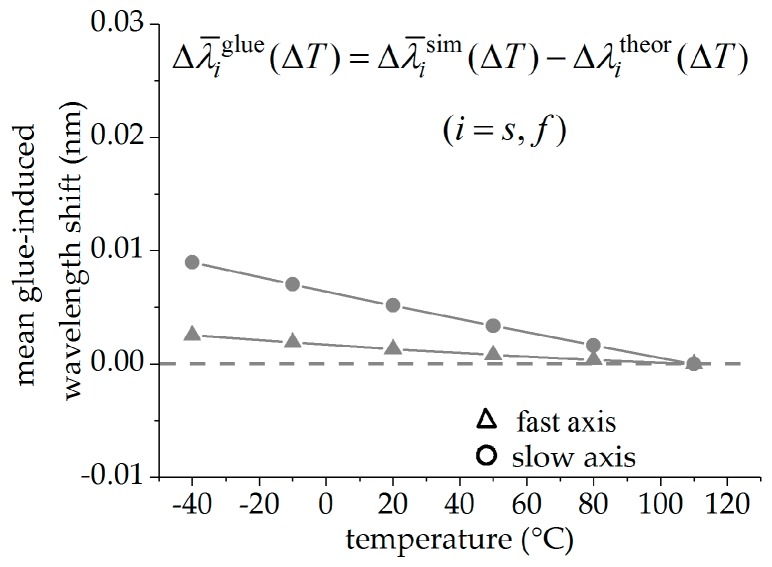
Glue-induced deviations of the mean values of the Bragg wavelength changes to the theoretical transversal stress-free FBG: Triangles mark values of the fast axis and circles of the slow axis.

The glue-induced Bragg wavelength shift $\Delta \lambda_{i,j}^{\mathrm{glue}}=\Delta \lambda_{i,j}^{\mathrm{sim}}-\Delta \lambda_{i}^{\mathrm{theor}}$ is given by the difference between the change in Bragg wavelength of the glued fiber $\Delta \lambda_{i,j}^{\mathrm{sim}}$ to the simulated values without glue-induced stress $\Delta \lambda_{i}^{\mathrm{theor}}$, and is plotted in Figure 3 for both fiber orientations ($j\in \{0^{{}^{\circ}},90^{{}^{\circ}}\}$) and both fiber axes (i∈{s,f}). Triangles mark the values of the fast axis, while circles mark the values of the slow axis. The values of the FBG in the 0°-orientation are plotted in blue, the values of the sensor in the 90°-orientation in green. The glue-induced wavelength shifts increase with decreasing temperature due to increasing glue-induced stress on the fiber. This effect is significantly higher in the slow axis compared to the fast axis in both fiber orientations. For the fast and for the slow axes, these deviations are nearly of the same value for both orientations but with the opposite sign. Figure 4 shows the glue-induced Bragg wavelength shift $\Delta {\bar{\lambda }}_{i}^{\mathrm{glue}}=\Delta {\bar{\lambda }}_{i}^{\mathrm{sim}}-\Delta \lambda_{i}^{\mathrm{theor}}$ for the averaged values of both FBGs (triangles for the fast and circles for the slow axis), which is supposed to be almost zero. For the fast axis, the maximal value of the glue-induced Bragg wavelength shift at −30 °C is significantly reduced from $\Delta \lambda_{90^{{}^{\circ}},s}^{\mathrm{glue}}=54\;\mathrm{pm}$ to $\Delta {\bar{\lambda }}_{s}^{\mathrm{glue}}=9\;\mathrm{pm}$ for the slow axis and from $\Delta \lambda_{90^{{}^{\circ}},f}^{\mathrm{glue}}=13\;\mathrm{pm}$ to $\Delta {\bar{\lambda }}_{f}^{\mathrm{glue}}=2\;\mathrm{pm}$ for the fast axis. Therefore, the mean values of the Bragg wavelength changes of both changes can be regarded as nearly independent of the glue-induced stress for both the signals of the fast and the slow axis, respectively.

## 4. Experimental: Surface-Attached Sensor Tandem

The capability of the above-described method in discriminating strain and temperature was investigated experimentally with an uncoated surface-glued PM tandem, which was attached to the surface of a spring steel bending beam. This setup was designed to apply strain between 0 με and 760 με to the fiber and could be used at different temperatures in a climatic chamber.

**Figure 5:**
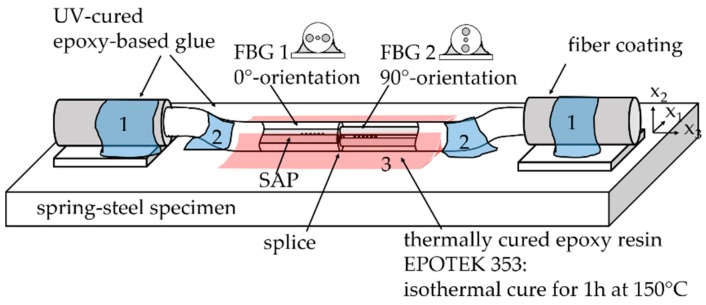
Sensor design with a surface-attached PM tandem: The fiber was azimuthally aligned with the slow axis of FBG1 and the fast axis of FBG2 parallel to the specimen’s surface and pre-fixed on the specimen with UV-cured epoxy resin at two points next to the FBGs (marked in blue). Afterwards, the entire sensing element was glued over a length of about 4 cm with thermal cured epoxy resin.

Figure 5 shows the used sensor design schematically. The FBG tandem consists of two FBGs near a splice in commercial Panda fibers (PM1550-XP, Nufern, East Granby, CT, USA). At the splice, the slow axis of the one fiber was aligned perpendicular to the slow axis of the other fiber. The splicing process was performed with a filament splicing unit (LFS4100, Thorlabs Vytran Europe, Exeter, GBR) which is capable of visually aligning the fibers before splicing, by capturing the cleaved fiber ends with a camera. The necessary removal of the coating for the splicing process lead to an uncoated sensor element of about 4 cm in length. An FBG was inscribed in 5 mm distance on each side (grating lengths 3 mm) of the splice, using our in-house inscription setup with a UV-laser and phase mask technique. To assure FBG wavelength multiplexing, phase masks with grating periods of 1074.8 nm and 1068 nm were used for the inscription of FBG 1 and FBG 2, respectively. The sensor element, which was previously aligned by analyzing the intensity pattern of the SAP with a microscope with lateral illumination, had been pre-fixed on steel platelets (marked with 1 in Figure 5) and was then attached to the surface of the bending beam (marked with 2 in Figure 5) with small drops of UV-curing epoxy resin. The sensor element was then glued over the whole length of about 4 cm with a thermally cured epoxy resin (EP353, Epoxy Technology Inc.: Billerica, MA, USA) under isothermal conditions at 150 °C for two hours (marked with 3 in Figure 5).

**Figure 6:**
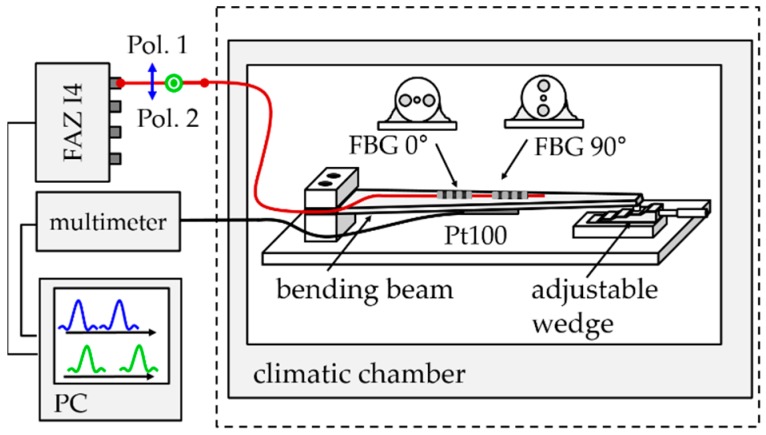
Measurement setup for temperature strain decoupling with surface-glued PM tandem sensor elements. PC, personal computer.

**Figure 7:**
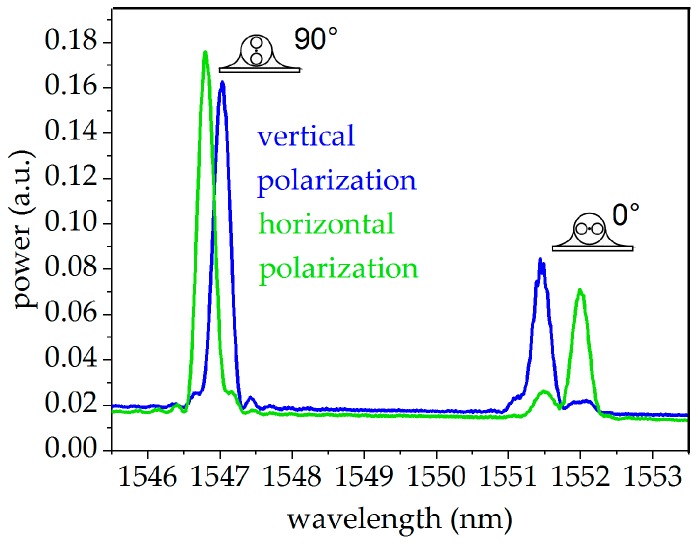
Spectra of the FBGs in the sensor tandem after the curing process at −30 °C.

The measurement of the Bragg wavelengths was performed with a high-resolution scanning laser interrogator (I4, FAZ Technology Ldt., Dublin, IRE) with a repeatability of 100 fm in the wavelength detection and a scanning frequency of 1 kHz [29]. The polarization state of the laser was switched between a horizontal and a vertical linear polarization after each scan and polarization was maintained in the entire measurement setup, including the fibers and the FBGs. The used measurement setup is shown in Figure 6. The corresponding spectra of the glued FBG, taken directly after the curing process at −30 °C, the temperature where high glue-induced stress was expected, are pictured in Figure 7 for both polarizations. The peaks of both polarizations are well-separated from each other while showing no sign of strong mode coupling, indicating the absence of strong glue-induced shear stresses.

The sensor element was attached on the top surface of a bending beam as depicted in Figure 6. External mechanical strain may be applied to the fiber by bending the beam with a wedge that provides five heights, leading to compressive strains between $\varepsilon_{3}^{\mathrm{mech}}=0\;\mu \varepsilon$ and $\varepsilon_{3}^{\mathrm{mech}}=760\;\mu \varepsilon$ in the FBGs. Strain cycles with increasing values up to −760 με and back to 0 με were performed at different temperatures, starting at 100 °C and going down to −30 °C in steps of 20 °C in a climatic chamber. Before starting a strain cycle, the temperature was kept constant for 1.5 h to assure isothermal conditions in the entire setup. At every strain step, the Bragg wavelengths were measured every second for five minutes and were averaged to eliminate fluctuations of the temperature control of the climatic chamber.

**Figure 8:**
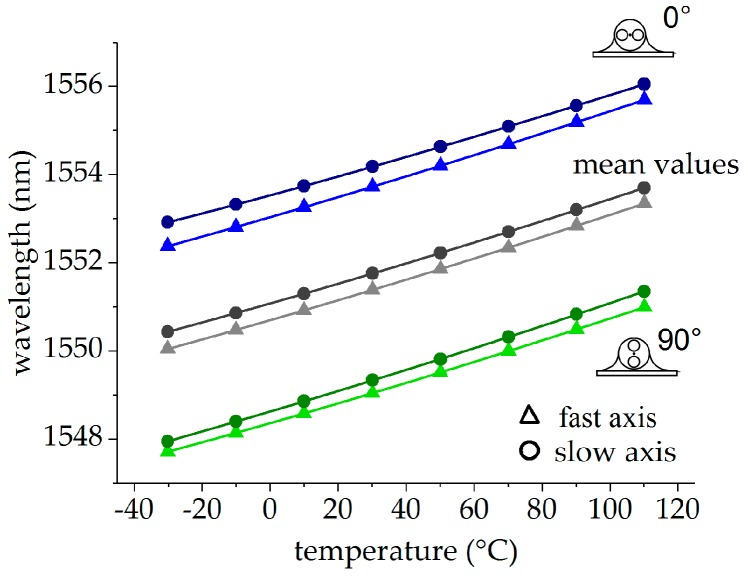
Temperature calibration of the surface-attached PM tandem: FBG in the 0°-orientation in green, FBG in the 90°-orientation in blue, and mean values in grey.

**Figure 9:**
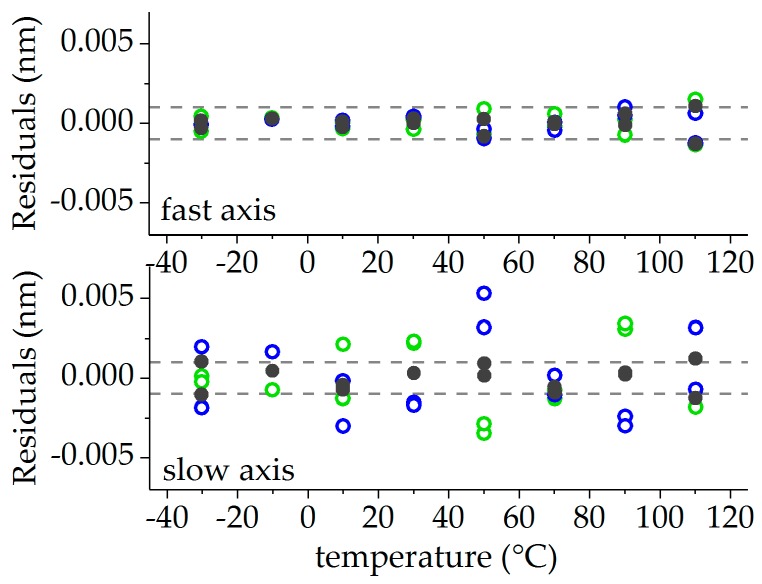
Residuals of the temperature calibration: FBG in the 0°-orientation in green, FBG in the 90°-orientation in blue, and mean values in grey.

All values without external mechanical strain were used for temperature calibration. The corresponding Bragg wavelengths $\lambda_{i,j}$ (with i∈{f,s} and $j\in \{0^{{}^{\circ}},90^{{}^{\circ}}\}$) for the slow and the fast axes of both FBG orientations (0°-orientation in blue, 90°-orientation in green) are shown in Figure 8. The mean values ${\bar{\lambda }}_{i}=0.5\left(\lambda_{i,0^{{}^{\circ}}}+\lambda_{i,90^{{}^{\circ}}}\right)$ taken from both orientations, which are the actual mesurands for temperature strain decoupling, are depicted in grey. Polynomial functions of the form

(9) $$\lambda_{i,j}=\lambda_{0,i,j}+a_{T,i,j}(T-T_{0})+a_{T^{2},i,j}{\left(T-T_{0}\right)}^{2}+a_{T^{3},i,j}{\left(T-T_{0}\right)}^{3}=\lambda_{0,i,j}+K_{T,i,j}(\Delta T)\cdot (T-T_{0})$$

with i∈{f,s} and $j\in \{0^{{}^{\circ}},90^{{}^{\circ}},\mathrm{mean}\}$ were fitted to all data and were also plotted in Figure 8. The fitting parameters are summarized in Table 2. The corresponding residuals, given as the difference of the measured wavelength and the fit, are plotted in Figure 9, with values originating from the FBG in the 0°-orientation depicted in blue, from the FBG in the 90°-orientation in green, and from the mean values of both FBGs in grey. The residuals of the fast axis scatter within a range of ±1 pm. In the slow axis, the residuals are significantly higher and in the range of ±5 pm. The residuals of the mean values of both sensors reduce the uncertainties for both the signals of the fast and the slow axis to values smaller than 1 pm for the whole temperature range between −30 °C and 110 °C. This indicates that the systematic uncertainties caused by glue-induced stress affect mainly the sensor signals of the slow axis in both FBGs and may be reduced by averaging the signals of both FBGs.

**Figure 10:**
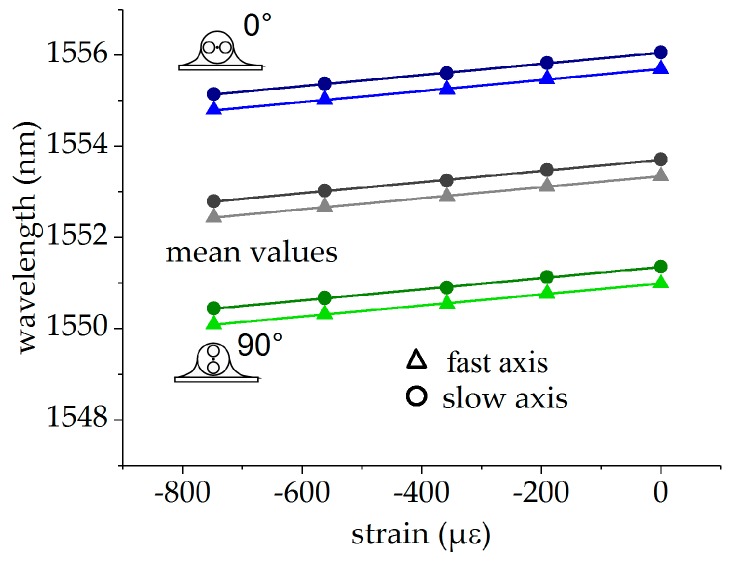
Strain calibration of the surface-attached PM tandem: FBG in the 0°-orientation in green, FBG in the 90°-orientation in blue, and mean values in grey.

**Figure 11:**
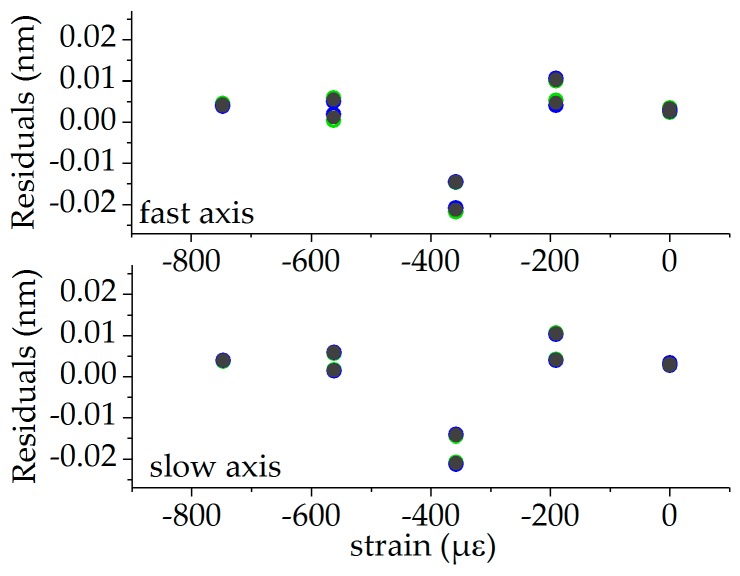
Residuals of the strain calibration: FBG in the 0°-orientation in green, FBG in the 90°-orientation in blue, and mean values in grey.

Strain calibration was performed at the reference temperature of 110 °C, since full strain transfer from the specimen to the fiber is assured, while significant glue-induced stress in the fiber can be excluded. The Bragg wavelengths for both fiber orientations and the mean values of both FBGs are plotted in Figure 10 (0°-orientation in blue, 90°-orientation in green, mean values in grey). The wavelength data of the single FBG and the mean values were fitted with a linear function, given by

$$\lambda_{i,j}=\lambda_{0,i,j}+a_{\varepsilon_{3},i,j}\varepsilon_{3}$$

with i∈{f,s} and $j\in \{0^{{}^{\circ}},90^{{}^{\circ}},\mathrm{mean}\}$. The corresponding fits are also depicted in Figure 10, and the fit parameters are summarized in Table 3. The residuals are shown in Figure 11, with values originating from the FBG in the 0°-orientation in blue, from the FBG in the 90°-orientation in green and from the mean values of both orientations in grey. In this case, the residuals show the same behavior for the fast and slow axis and in both orientations and the averaged values of both orientations. This indicates that these deviations are probably caused by systematic uncertainties in the height of the wedge, which was used to introduce the strain by bending the cantilever. This limits the accuracy of the strain measurement.

## 5. Results and Discussion: Strain-Independent Temperature Measurement

**Figure 12:**
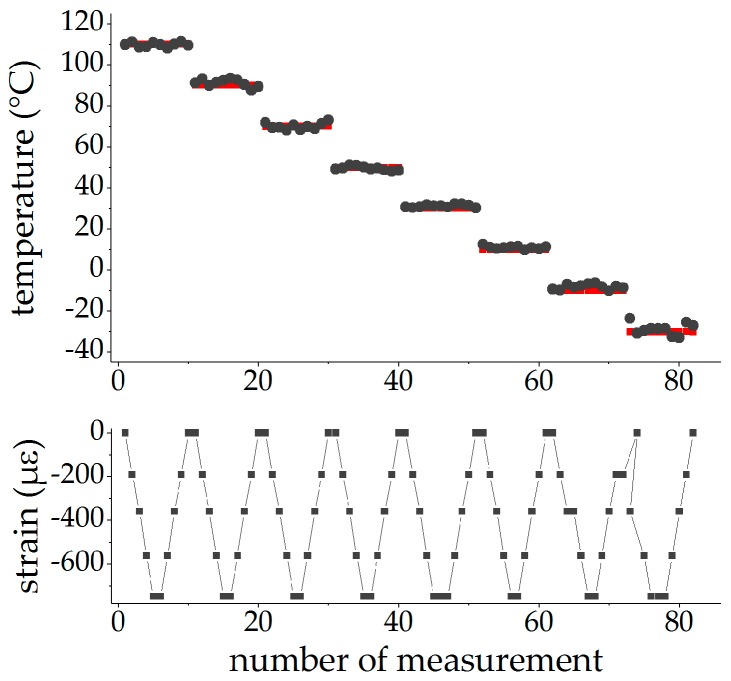
Strain-independent temperature measurement with surface-glued FBGs: upper graph: evaluated temperature (black dots) in comparison to the reference temperature of the Pt100 sensor (red), lower graph: applied strain.

Using the averaged changes in the Bragg wavelengths $\Delta {\bar{\lambda }}_{i}$ (with i∈{f,s} for the fast and slow axes) of both FBGs of the PM tandem enables the measurement of temperature in the presence of external mechanical longitudinal strains with surface-glued FBG tandems. The results of the temperature determination after five iteration steps with the matrix approach of Equation (7) is shown in Figure 12 marked with black dots.

The matrix elements ${\bar{K}}_{T,i}^{\mathrm{gl}}(\Delta T)=K_{T,i,\mathrm{mean}}(\Delta T)=a_{T,i,\mathrm{mean}}+a_{T^{2},i,\mathrm{mean}}\Delta T+a_{T^{3},i,\mathrm{mean}}\Delta T^{2}$ for the temperature and ${\bar{K}}_{\varepsilon_{3},i}=a_{\varepsilon_{3},i,\mathrm{mean}}$ for the strain sensitivity were taken from the fits to the mean values, as given in Table 2 and Table 3 (mean values). The reference temperature, measured with the Pt100 sensor, is depicted in red and the corresponding strain values are shown in the lower graph in Figure 12.

**Figure 13:**
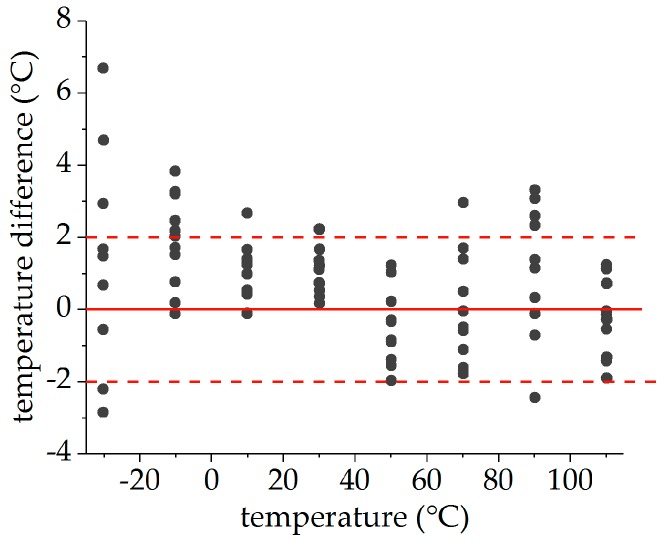
Deviations of FBG temperature to reference temperature.

The temperatures evaluated with the FBG tandem matched the reference values independently of the applied strain over the entire temperature range between −30 °C and 110 °C. Figure 13 shows the deviation of the evaluated temperatures from the reference temperatures of the Pt100 sensor in dependence of temperature. With exception of the measurement at −30 °C, all deviations stay within a range of ±4 °C around the reference temperature. Larger deviations (up to 7 °C) have only been observed at the lowest temperature and could probably be attributed to the high glue-induced wavelength changes of about 150 pm at this temperature. Due to the small differences in the sensitivity constants, the suppression of these deviations by using the mean values have to be achieved within the picometer range. Given that the glue was suspended by hand, resulting in a variable thickness of the applied glue along the fiber, these are good results that prove the capability of this method.

## 6. Conclusions

Strain-compensated temperature measurements have been successfully realized within a wide temperature range between −30 °C and 110 °C with surface-glued PM-FBG sensor elements for the first time, using azimuthally aligned FBG tandems consisting of two FBGs in PM fibers that were spliced with their optical axes perpendicular to each other. The main advantage of this novel methodology lies in the fact that glue-induced transversal forces could be compensated for during the evaluation process by averaging the Bragg wavelengths of the fast and of the slow axes of both PM-FBGs in the surface-glued FBG tandem. The capability to compensate for glue-induced transversal forces of varying strength have been shown for temperature-dependent glue-induced transversal stress introduced during temperature changes as a result of the high CTE of the epoxy resin. This measurement technique enabled temperature and strain decoupling with an uncertainty of several degrees in the entire measurement range. We expect a similar compensation capability for all glue-induced transversal forces that are based on volume changes of the used glue, e.g., water absorption, which will be addressed in further research. This technique for strain and temperature decoupling with surface-attached FBG tandems is therefore of great importance for all applications in which fully glued fibers are essential. Additionally, the utilization of the FBG tandem approach to embedded fiber optic multi-parameter sensing will be addressed in upcoming research, which further extends the application range of such tandem elements.

## 7. Patents

The functional working principle and the corresponding sensor design are registered at the German Patent and Trademark Office (Deutsches Patent-und Markenamt, DPMA): DE 10 2017 201 523.3 (Faseroptische Erfassungseinrichtung sowie Verfahren zum Betreiben einer solchen faseroptischen Erfassungseinrichtung), 31.01.2017.

## Figures and Tables

**Table 1:** The Material Parameters used for mechanical and optical simulation.

Section	Young’s Modulus (GPa)	Poisson’s Ratio	CTE (·10^−6^ °C^−1^)	Refractive Index	Lit.
Glue	3	0.33	54	-	[26,27]
Fiber cladding	72	0.16	0.54	1.444023	[24]
Fiber core	71	0.16	0.95	1.449423	[24]
SAP	42	0.20	2.38	1.444023	[24]

SAP, stress-applying part; CTE, coefficient of thermal expansion.

**Table 2:** Fitting parameters of the temperature calibration of the PM tandem.

Orientation j	Axis i	$\lambda_{0,i,j}$ nm	$a_{T,i,j}$ pm/°C	$a_{T^{2},i,j}$ ·10^−2^ pm/°C^2^	$a_{T^{3},i,j}$ ·10^−6^ pm/°C^3^
90°	s	1551.3540	26.54	1.59	−2.84
	f	1550.9939	25.36	1.04	−2.67
0°	s	1556.0518	24.39	1.00	−3.32
	f	1555.6985	25.64	1.08	−2.33
mean	s	1553.7029	25.46	1.30	−2.84
	f	1553.3462	25.50	1.04	−2.67

**Table 3:** Fitting parameters of the strain calibration of the PM tandem.

Orientation j	Axis i	$\lambda_{0,i,j}$ nm	$a_{\varepsilon_{3},i,j}$ pm/με
90°	s	1551.3522	1.22
	f	1550.9924	1.21
0°	s	1556.0523	1.22
	f	1555.6981	1.21
mean	s	1553.7023	1.22
	f	1553.3452	1.21
